# Mechanotransduction-Induced Lipid Production System with High Robustness and Controllability for Microalgae

**DOI:** 10.1038/srep32860

**Published:** 2016-09-09

**Authors:** Myung Kwon Cho, Hwa Sung Shin

**Affiliations:** 1 Department of Biological Engineering, Inha University, Incheon, 402-751, Korea

## Abstract

Microalgae lipids are a promising energy source, but current biochemical methods of lipid-inductions such as nitrogen deprivation have low process robustness and controllability. Recently, use of mechanotransduction based membrane distortion by applying compression stress in a 2D-microsystem was suggested as a way to overcome these limitations of biochemical induction. However, reproduction in large numbers of cells without cell death has been difficult to overcome because compression for direct membrane distortion reduces culture volume and leads to cell death due to nutrient deprivation. In this study, a mechanotransduction-induced lipid production (MDLP) system that redirects elastic microbeads to induce membrane distortion of microalgae with alleviating cell death was developed. This system resulted in accumulation of lipid in as little as 4 hr. Once compressed, porous microbeads absorb media and swell simultaneously while homogeneously inducing compression stress of microalgae. The absorbed media within beads could be supplied to adjacent cells and could minimize cell death from nutrient deficiency. All mechanotransduction was confirmed by measuring upregulation of calcium influx and Mat3 genes. The microbeads ensured robustness and controllability in repeated compression/de-compression processes. Overall, the MDLP system has potential for use as a fundamental biodiesel process that requires robustness and controllability.

Photosynthesis is a representative example of organic conversion in the environment^1^. Microalgae, which have the capacity for mass storage of TAG (triacylglycerol), are a suitable model of organic conversion^2^. The unicellular green algae *Chlamydomonas reinhardtii* has been used to investigate various fundamental biological processes, including lipid metabolism^3^. Studies have recently been conducted to identify factors, inducing organic conversion, for optimization of lipid production^4^. These factors include chemical and environmental cues such as nitrogen deprivation, temperature and pH stresses^5^,^6^,^7^. However, these conventional stresses have a limitation. Their factors have relatively low conversion rate compared to mechanical stress, ranging from one day to as high as over^5^,^6^,^7^,^8^. Moreover, long-term stress could have negative effects on cell viability and growth, leading to low lipid production efficiency^9^,^10^. More importantly, media conditions readily change during cell culture, which aggravates the process, leading to low robustness and controllability.

Mechanical stresses are possible cues that trigger biological metabolism^11^. Some studies have revealed that shear stress and compression stress lead to changes in microalgal metabolism^8^,^12^. This is especially true for stress induced changes in lipid metabolism, which have been actively studied and shown to occur via mechanotransduction transmitted from the membrane distortion of the cell surface^13^. However, hydrodynamic stresses such as shear stress transmit mechanical signaling indirectly to cells through medium, resulting in difficulty in controlling the cell membrane distortion in the temporal aspect and intensity of stress. We recently showed that direct compression-induced membrane distortion caused microalgae to very rapidly synthesize free fatty acids and convert them into TAG in a microfluidic device^8^. The compression inducer is very robust and controllable because the stress does not vary and is easily manageable, unlike chemical inducers. However, a scaled-up system must be developed to enable production of free fatty acids, lipids or biodiesel on an industrial scale.

This study was conducted to develop a conceptual large-scale system to enable compression-based synthesis of free fatty acids and high-speed conversion into TAG. Polyurethane is an elastic polymer. The elastomeric beads could be fabricated from this polyurethane elastomer. The fabricated polyurethane beads had a specific physical property that beads were easily compressed and restored to their original form when the compressive force was removed. Using this restoring force of beads, it could give a compressive stress to the cells indirectly. In other words, when the compressed beads were restored in limited spatial extent, the cells adjacent to the beads got a compressive force. The highly elastic polyurethane beads made it possible to exert compressive stress, triggering lipid metabolism without cell loss and death during the process.

## Results

### Design of the MDLP (Mechanotransduction-induced lipid production) system and process for direct membrane distortion of *C. reinhartii*

**Figure 1:**
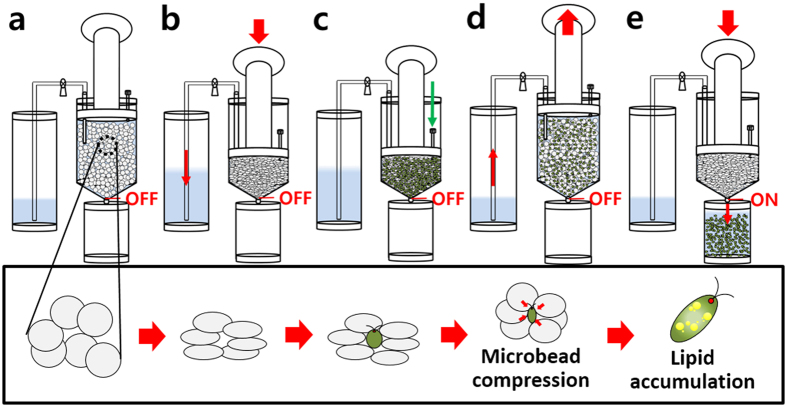
Design and fabrication of MDLP (Mechanotransduction-induced lipid production) system. Overall description of process design and components. (**a**) Initial state. (**b**) Microbeads compression. (**c**) Inoculation of microalgae. (**d**) Microbeads de-compression for induction of mechanotransduction to microalgae. (**e**) Microalgal harvesting.

**Figure 2:**
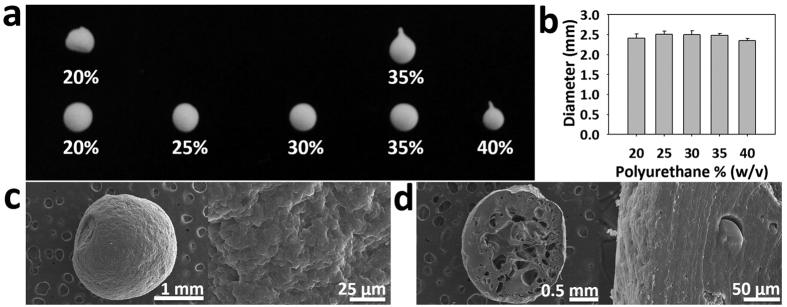
Optimization of elastic microbeads used to induce mechanotransduction of microalgae. The effects of PU density on (**a**) microbead morphology and (**b**) size. (**c**) SEM images of porous microbead surface and (**d**) inside of the microbead. The density of the PU beads was 30% (w/v) of the PU concentration.

The MDLP system was designed by applying the principle of mechanotransduction that the lipid accumulation was induced by membrane distortion of the cell, and then fabricated to induce the organic conversion of microalgae (Fig. 1). When the compressed beads and microalgae were mixed in the reactor, the inflated beads occupied the pre-vacant space, which transmitted compressive force to cells equally via the spatial dispersion of the force. Operation of the prototype device was confirmed based on the finding that it went through the successful process of compression, inoculation, de-compression and harvest step. About 60 × 10^6^ cells were seeded in one cycle of process. If cell is pre-seeded and compressed, lots of cells would be expelled with the media. This is why the beads were first compressed and then, cells were seeded and compressed by the inflating bead force. The spherical and elastic polyurethane beads became distorted by the compression force and were restored by absorbing surrounding media. SEM images were analyzed to understand the physical properties of this PU bead. As shown in Fig. 2c, there were countless microscopic pores on the surfaces of the beads, which allowed liquids to enter and exit the pores of the beads. There were also empty spaces within the bead that could absorb a certain amount of liquid. These findings confirmed that the prototype of the device could actualize the process shown in Supplementary Fig. S1.

### Optimization of polyurethane bead size and restoring force

The fabricated shape of the bead was analyzed according to the polyurethane concentration (Fig. 2a). Generally, secondary distilled water was used to solidify the polyurethane droplets. Since 20% PU is less dense than water, it could not form a bead and burst on the surface of the solvent. Therefore, the density of the solvent was reduced by changing the solvent from distilled water to 30% ethanol to address this problem, after which the shape of the fabricated bead was analyzed. The 20% PU bead was very unstable because of the low density. The fabricated shape was irregular and the bead shrank in response to the water pressure. The 35% and 40% PU beads had tails because of the high viscosity of the PU solution. The 25% and 30% PU beads were confirmed to be fabricated in the form of spheres of uniform size. To compare the size of beads relative to the PU concentration, 20 beads in each concentration were selected at random and their diameters were measured. As shown in Fig. 2b, the bead size did not differ relative to the PU concentration, confirming that the PU concentration does not affect the bead size. A PU concentration of 30% was selected as the optimal concentration based on the above results and its strong durability and elasticity by comparison with 25% PU beads.

### Calcium influx-mediated deflagellation and cell size variation under bead compression stress

**Figure 3:**
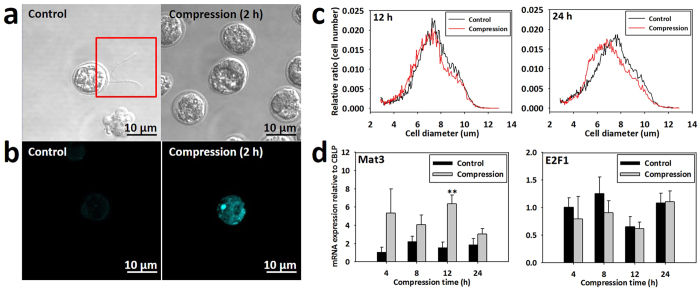
Verification of microbeads-induced mechanotransduction in view of calcium influx, deflagellation and cell size. Bead compression invoked (**a**) deflagellation and (**b**) calcium influx to microalgae. Bead compression decreased (**c**) microalgal size and increased (**d**) its associated gene, Mat3. T-test of P values was conducted according to control and compression (*P < 5.0×10^−2^, **P < 1.0×10^−2^, ***P < 1.0×10^−3^).

*C. reinhardtii* was deflagellated by bead compression stress and the Ca^2+^ influx was greater in the compressed cell than the control cell. (Supplementary Fig. S2 and Fig. 3a,b). The size of compressed cells harvested from the device was compared with that of control cells (Fig. 3c). The results showed that the average size of the compressed cells decreased relative to the control cells. In the case of cells that were compressed for 12 hours, the largest cell diameter was 7.2 μm. At less than 7.2 μm, the compressed cells showed greater distribution than the control cells. After compression for 24 hours, the uncompressed and the compressed cell had a maximum size at 7.8 μm and 6.7 μm, respectively. The overall compressed cell diameter decreased by 0.5 to 1 μm compared with the uncompressed cells. Based on these results, bead compression stress induced membrane distortion, followed by increased Ca^2+^ influx into the cell, deflagellation, and small cell size. The mRNA of Mat3 and E2F1 were related to these physiological changes^14^. As shown in Fig. 3d, Mat3 mRNA was upregulated in all compression samples, while there was no tendency to upregulate in E2F1 mRNA.

### Bead compression stress upregulated lipid accumulation in *C. reinhardtii*

**Figure 4:**
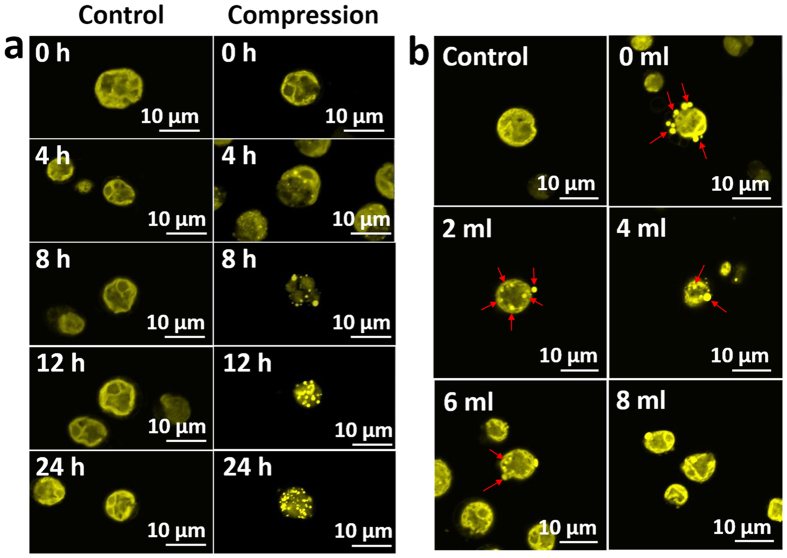
Effect of microbeads mechanotransduction on lipid synthesis of microalgae. (**a**) Nile red staining of lipid droplets in microalgae with respect to compression times. (**b**) Nile red staining of lipid droplets in microalgae with respect to compression levels.

**Figure 5:**
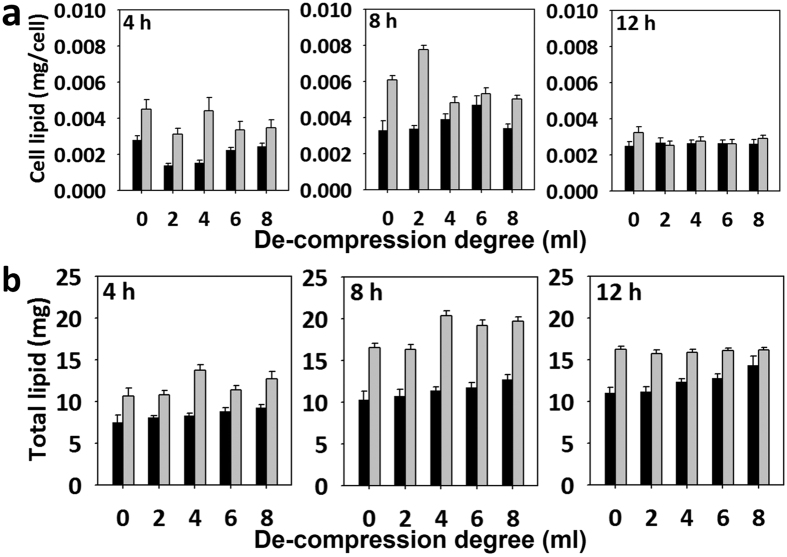
Quantitative analysis of lipid synthesis of microalgae under microbead compression. Black bar is a control and gray bar is a compressed sample. (**a**) Unicellular synthesis of lipid droplets with respect to compression times and compression levels. (**b**) Total synthesis of lipid droplets with respect to compression and times of compression.

The membrane distortion of microalgae induced by mechanical stress resulted in increased lipid accumulation in the cell^8^,^12^,^15^. After subjecting cells to bead compression stress, qualitative and quantitative analysis was conducted every 4 hours to determine if lipid droplets occurred in the cell. As shown in Fig. 4a, lipid droplets were observed in compressed cells, starting at 4 h of compression, which was the minimum compression time required to induce lipid accumulation in *C. reinhardtii* (Supplementary Fig. S3). The lipid droplet size and number increased with compression times. In addition, the accumulation of lipid droplets according to compression levels was analyzed (Fig. 4b). Compression levels were controlled by a de-compression degree of syringe and the compression time was fixed for 12 h. Lipid droplets were observed up to the 6 ml de-compression degree. The size and number of lipid droplets was significantly decreased in 6–8 ml de-compression sample. Next, following the quantitative results by a sulpho-phospho-vanillin assay, the amount of lipids per unit of compressed cells was higher than that per unit of uncompressed cells in all de-compressed samples at 4–8 h of compression (Fig. 5a). However, 12 h of compression showed similar phenomena only in the 0 ml de-compression sample. Total lipid amount was also higher in compressed cells at all 4-12h of compression (Fig. 5b).

### Optimization of lipid production efficiency under bead compression stress condition

To maximize lipid production efficiency, two of the most critical conditions, compression time and the degree of compression force, were optimized. The degree of compression force was adjusted by de-compressing a piston of the syringe in 2 ml increments and then analyzing the samples every 4 h. As shown in Fig. 5b, the total lipid content of the de-compressed sample was higher than that of the uncompressed sample at all compression times. The highest amount of total lipids was measured in the 4 ml de-compression sample for each compression time. When only compression time was evaluated, 8 h resulted in the highest total lipid content in 4–8 ml de-compression samples. Consequently, the 8 h compression time/4 ml de-compression sample showed the highest lipid production efficiency.

### Changes of lipid contents in *C. reinhardtii* under bead compression stress

**Figure 6:**
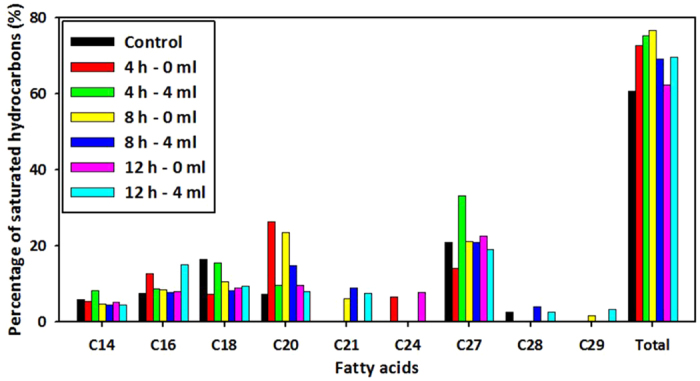
Percentage of saturated hydrocarbon in total hydrocarbon. C14 (Tetradecane), C16 (Hexadecane), C18 (Octadecane), C20 (Eicosane), C21 (Heneicosane), C24 (Tetracosane), C27 (Heptacosane), C28 (Octacosane), C29 (Nonacosane) and total is sum of C14-29 saturated hydrocarbon. Seven different samples were analyzed in accordance with conditions of compression time (4, 8, 12 h) and compression levels (0, 4 ml).

Lipid contents of *C. reinhardtii* according to bead compression was analyzed by GC/MS. Seven different samples were prepared in accordance with conditions of compression time (4, 8, 12 h) and compression levels (0, 4 ml) and their lipid contents were analyzed (Table 1). Specific changes of lipid contents were rearranged in respect of biofuel (Fig. 6). A hydrocarbon converted in microalgae is a source of second and third biofuels^16^. Saturated hydrocarbons, alkanes, are the fundamental source of petroleum fuels^17^. Especially, long chain saturated hydrocarbon is related to the higher heating value (HHV) which is a representative property defining efficiency of fuels^18^. All compressed samples showed that ratio of total saturated hydrocarbons to total hydrocarbons increased as compared with the control.

### Analysis of lipid-related mRNAs in *C. reinhardtii* under bead compression stress

**Figure 7:**
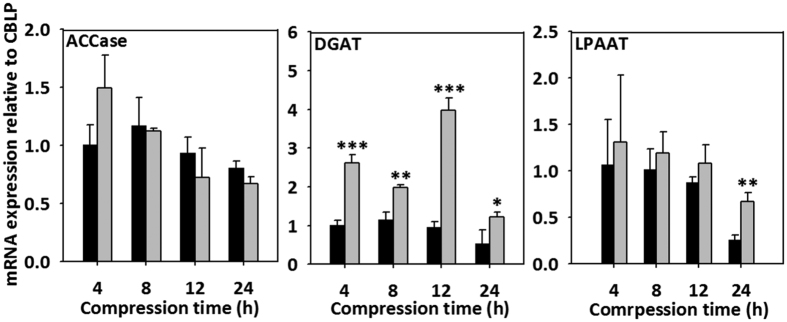
Upregulation of lipid-related mRNAs in *C. reinhardtii* under microbead compression. A t-test of P values was conducted according to control and compression (*P < 5.0 × 10^−2^, **P < 1.0 × 10^−2^, ***P < 1.0 × 10^−3^).

A previous study confirmed that lipid-related mRNAs in *C. reinhardtii* were upregulated under compression stress^8^. The MDLP system developed in this study as a scaled-up version of the previously described system showed similar results, with mRNA of ACCase, DGAT and LPAAT, which are known to be associated with free fatty acid and TAG synthesis, being upregulated. As shown in Fig. 7, two mRNAs were upregulated at every compression time, except ACCase, which was upregulated at 4 h of compression, then gradually decreased as the compression time increased.

### Maintenance of cell viability under limited culture environments

**Figure 8:**
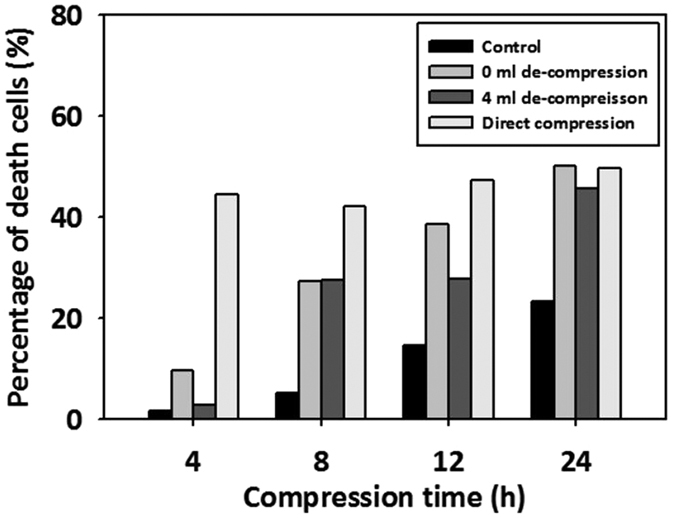
Cell death of microalgae under microbead compression stress. The percentage of death cells was calculated by (the number of SYTOX green stained cells)/(the number of total cells) × 100 = Percentage of death cells (%).

To determine if the viability of cells was maintained under bead compression stress, compressed cells were stained with 1 μM SYTOX green and their microscopic images were taken using a confocal microscope. Each control, non-decompression, and 4 ml de-compression sample was observed at different compression times (4, 8, 12, 24 hours). As shown in Fig. 8, all direct compression samples had high percentage of death cells over 40%. The percentage of death cells increased over time in the non-decompression sample. However, 4 ml de-compression sample samples showed relatively low percentage of death cells in all compression times except 24 h compression time. As a result, the 4 ml de-compression sample showed that the cell viability was maintained under optimized compression stress environments.

### Robustness of the MDLP (Mechanotransduction-induced lipid production) system

The most critical factor in determining the robustness of the MDLP system is the elasticity of the PU bead. The physical properties of the bead such as size and elasticity were assessed through repeated compression experiments to verify the robustness. The bead size did not change with repeated experiments. Conversely, the elasticity of the bead gradually decreased, but not to the certain elastic force and was maintained continuously (Supplementary Fig. S4a). However, the lowered elasticity did not influence the induction of lipid formation (Supplementary Fig. S4b), which implies that the diminished elasticity of the bead was sufficient to compress the cell for lipid accumulation. At the uncompressed step, the acceleration of dispersion time of the reused bead had no effect on lipid production efficiency. The surface color of the bead became green when it was co-cultured with *C. reinhardtii* over one week. These findings verified that the green pigment of *C. reinhardtii* was released by cell death or membrane distortion and absorbed onto the surface of the bead (Supplementary Fig. S4c), confirming that the phenomenon of the discoloration did not affect lipid production.

## Discussion

Direct membrane distortion was shown to stimulate lipid conversion of microalgae under a microfluidic scale^8^. However, before this phenomenon is extended to a larger scale, several problems must be addressed. The process should have no cell loss and minimize cell death during compression. Additionally, it is important to transfer the spatially homogenous and compressive stress. Traditional compression systems such as compressive loading systems do not meet the above three requirements^19^. Therefore, we propose the MDLP (Mechanotransduction-induced lipid production) system, which meets all of the aforementioned requirements (Fig. 1).

The three requirements can be resolved as follows. The elastic feature, empty space in the interior and semipermeable surface of the bead enable a certain amount of liquid to be soaked into the bead. These features prevent the compressed microalgae from being under nutrient-deficient stress with the help of nutrients released from the bead pores. If compressed in a normal direction on a plane, the cells must have received the nutrient deficiency and compression stress simultaneously, because it is difficult to re-supply nutrients to the cells (Supplementary Fig. S5), resulting in low viability before lipid accumulation (Fig. 8). On the other hand, bead compression can reduce nutrient deficiency stress because it is possible to supply medium continuously through the adaptation bead that is holding a certain amount of TAP medium inside. Moreover, the 2D compression system cannot help to have low storage efficiency for suspension cells due to the leaking-out cells when compressed in a normal direction (Supplementary Fig. S5). Conversely, the microbead system can secure a certain amount of cells owing to the three-dimensional working volume. Finally, soaking media inflates the volume of the compressed bead, which exerts compression stress on cells by reducing the volume pre-occupied by the cells. Based on the above process, spatially homogeneous compressive stress must be transmitted to the cells because homogeneous beads are used and a hydrostatic press is equally-distributed in the three-dimensional space. Overall, the MDLP system can homogeneously compress each of suspended cells (About 60 × 10^6^ cells at one process) and minimize cell loss without immobilization or utilization of a micron membrane with a clogging risk. As a result, the novel MDLP system is a highly-controllable method adequate for application to suspensions of cells.

Mechanotransduction-driven lipid synthesis was observed in the scaled-up device. The cellular physiology was analyzed at the transcript-level to examine whether the scaled-up novel MDPL system was synthesizing high amounts of lipids. First, the scale-up device successfully induced mechanotransduction to the compressed cells. A previous microfluidic study confirmed that compression stress-induced elevation of the intracellular Ca^2+^ caused deflagellation of *C. reinhardtii*, which is relevant to severing of the axonemal microtube via a Ca^2+^-mediated contraction of a stellate array of centrin-containing fiber in the transition zone^20^,^21^. Similarly, application of bead compression induced Ca^2+^ influx and invoked deflagellation. Small cell size and upregulation of Mat3, which are other markers for compressed microalgae^8^, were confirmed in bead compression. However, discrete multiple fission was not observed, unlike in the microfluidic device^8^. This is thought to be because the compression level was lower than that of the microfluidic device. Although the method of applying the compression was different, the compression system was successfully scaled-up. Evaluation of the lipid metabolism revealed that more lipid drops were synthesized by bead compression and the mRNA expressions of LPAAT, and DGAT increased except ACCase. ACCase was upregulated only at 4 h of compression, then gradually decreased as the compression time increased, but DGAT was remarkably upregulated. ACCase facilitates the biotin-dependent carboxylation of acetyl-CoA to produce malonyl-CoA, which generates the biosynthesis of fatty acids^22^. LPAAT facilitates the acylation of sn-1-acyl lysophosphatidic acid (lysoPA) to form phosphatidic acid, which is the common part of the phospholipid^23^. In addition, DGAT is a key regulator facilitating the formation of triglycerides by the combination of diacylglycerol and a third acly-CoA^24^. These three regulators increase the free fatty acid in the chloroplast. In cytosol, they induce organic conversion as TAG by facilitating the transformation from PtdOH to DAG and from DAG to TAG^3^. As well as lipid, long-chain saturated hydrocarbon, which was considered to be more synthesized in response to self-defense mechanism under bead compression^25^, making MDLP a promising system for biofuel production since it can also be transformed to biofuel.

The most important condition of compression stress devices on an industrial scale is robustness. The robustness of lipid productivity was confirmed based on measurement of bead performance as the process was repeated 10 times (Supplementary Fig. S4). Even though bead elasticity was reduced and pigment-colorizing progressed in the beads during repeated experiments, the beads were able to induce lipid conversion of microalgae.

Lipid production through organic conversion in microalgae has great potential for use as a next generation biofuel. Biochemical conversion factors such as nitrogen deprivation, temperature, pH, and osmotic pressure have been investigated to determine if they can induce high lipid synthesis. However, mechanical conversion factors such as compression, stretching and fluidic shear stress are promising in terms of controllability and robustness because the biochemical conversion factors are hindered by uncontrollability and a lack of robustness. Overall, these findings indicate that bead compression is a progressive method that not only has the advantage of a mechanical conversion factor, but also the potential to be scaled-up for industrial applications.

This study was conducted to develop a system with high robustness and controllability that can induce lipid conversion via application of direct membrane distortion to massive microalgae. The simultaneous and homogenous direct membrane distortion of microalgae was induced by the elastic restoration force generated via absorption of nutrient broth by the porous PU microbead. Moreover, nutrients in beads prevented cell death. The mechanotransduction of cells was induced by mechanical stress and transcript signaling was accomplished via calcium ion influx. Eventually, the expression of free fatty acid and TAG related genes was upregulated. With this system, the compressed microalgae showed increased lipid droplets and long-chain hydrocarbon without cell death. Compression of microalgae for 4 hours induced an increase in lipid production of 1.65 times, while compression for 8 hours led to an increase of 1.78 times relative to the control. From an economic perspective, the MDLP system required more cost in comparison with conventional biochemical system. However, the induction time to attain the maximum of lipid production was much shorter than conventional biochemical system, which enabled the much improved productivity. As shown in Supplementary Table S1, the MDLP system had the high lipid productivity compared with conventional biochemical systems. Despite containing initial process installation and operating costs, the economic profits can be expected to increase through high productivity. For this reason, the cost implication is guaranteed. In addition, the ease of scale-up about the MDLP system was proved in Supplementary Fig. S6. In conclusion, this system overcomes the limitations of the existing biochemical induction system, which has low robustness, low controllability and a long induction time. Furthermore, this fast lipid conversion system has high applicability for the development of microalgae biodiesel systems.

## Methods

### MDLP (Mechanotransduction-induced lipid production) system design and fabrication

The MDLP system is composed of three parts, a compression reactor, feed tank, and a harvest tank (Fig. 1). The compression reactor is filled with fabricated polyurethane (PU) (Cardio Technology International, Japan) beads. The process is operated in a mode of compression, inoculation, de-compression, and harvest progressively (Fig. 1). In the case of the compression step, a certain amount of the medium is expelled through a stainless steel conduit located at the top of the reactor. After the bead is completely compressed, cell seeding is performed through the inoculation site. In the de-compression step, as soon as the compression force is released, the media of the feed tank is charged back into the compression reactor and absorbed inside the beads. After agglomerate beads are restored by fed media, the feed tank conduit is blocked. In this step, microalgae are subject to compression stress via the elasticity of PU beads. Finally, cells are collected in the harvest tank through recompression of the piston. To reproduce the above scheme, the prototype of the MDLP system was manufactured. As shown in Supplementary Fig. S1, the compression reactor was made based on a 50 ml syringe (Henke-Sass, Wolf, Germany). A hole was made in the plunger part of the syringe and an 18G needle (Hamilton, Switzerland) was inserted into the hole. The needle, which was fixed using a UV bond (Norland Product, USA), was connected with a 3ml syringe (Henke-Sass, Wolf, Germany) that played a role as a feed tank using standard silicone tubing (I.D. 1.5, O.D. 2.5 mm) (Daihan Scientific, Republic of Korea). The harvest site was tip of the 50 ml syringe (Henke-Sass, Wolf, Germany).

### Bead fabrication and characterization

PU is an elastomeric polymer that is used as bead material. The PU template was made to 30% (w/v) PU solution in N,N-dimethylformamide (DMF) (Junsei Chemical, Japan). The PU solution was dissolved overnight, then loaded into a 20 ml plastic syringe (Henke-Sass, Wolf, Germany) connected to a 22 gauge stainless steel needle (Hamilton, Switzerland). The syringe sets were installed in a syringe pump (KD Scientific, USA) with a flow rate set to 3 ml/h. PU beads fell into the sphere from the syringe and were collected in distilled water. The freefall height from the syringe tip to the surface of the distilled water was maintained at 9 cm. Fabricated PU beads were soaked in 95% ethanol (Samshun Pure Chemical, Republic of Korea) overnight to remove the residual DMF in beads. After washing three times in distilled water, beads were soaked in TAP media (Gibco, USA) overnight for adaptation. During this process, beads were compressed repeatedly to remove the internal liquid. The morphology of the fabricated PU bead was observed by scanning electron microscopy (SEM) (S-4200, Hitachi, Japan). Before conducting SEM, the sample was coated using a Pt sputter for 180 seconds. Finally, the SEM image of the PU bead was obtained at a 10 kV accelerating voltage of the SEM. In addition, the change in bead diameter in accordance with the compression time was measured using a micrometer to identify the bead elasticity and robustness.

### Culture of *Chlamydomonas reinhardtii*

The wild type of *C. reinhardtii* CC-621 (Chlamydomonas Resource Center, USA) was seeded in 18 ml TAP media at pH 7.2 (Gibco, USA) in a 50 ml Erlenmayer flask (Duran, Germany). The flask was then cultured in a shaking incubator (5% CO_2_, 120 rpm) at 23 °C under 300 μm·m^−2^·s^−1^ that was maintained using two OSRAM DULUX L FPL36EX-D lamps (Osram, Germany). Alternating periods of light and dark (12 h/12 h) were provided to synchronize cell growth and division. After three cycles, synchronization was verified. The cell optical density was measured using a spectrophotometer (Sunrise^TM^, Tecan, Switzerland) to ascertain whether or not synchronization had occurred and a growth curve was generated. The change in the cell growth curve was monitored for three light/dark cycles. When the fourth period started, the inoculation density of the synchronous cell was adjusted to 10–12 × 10^6^ cells/ml. Finally, 5 ml of inoculation solution were seeded into the MDLP system containing polyurethane beads.

### Viability and cell death of *C. reinhardtii*

The sample from the reactor was stained using 10 μM SYTOX Green (Molecular Probes, USA) in TAP media for 10 minutes at room temperature. Live cells in the sample were observed at 488 nm (Ar-laser)/505-530 nm (excitation/emission) and dead cells were observed at 543 nm (HeNe-laser)/560 nm (excitation/emission) using a confocal laser microscope (LSM 510 META, Carl Zeiss, Germany). The ZEN 2009 Light Edition software (Carl Zeiss, Germany) was used to merge dual fluorescence images. About 500 cells in a random field at 100×  magnification was utilized to calculate the cell death of *C. reinhardtii*.

### Cell morphology

To compare the morphologies of compressed and uncompressed cells, each sample was centrifuged at 13,500 g for 20 seconds, then fixed using 0.25% glutaraldehyde solution (Sigma Aldrich, USA). The cell membrane distribution and deflagellation were subsequently observed by confocal laser microscopy (LSM 510 META, Carl Zeiss, Germany) and the cell size was measured using a Cellometer Auto T4 Cell Counter (Nexcelom Bioscience, USA) (n > 50).

### Identification of cytosolic free Ca^2+^ influx

*C. reinhardtii* samples from the reactor were centrifuged at 13,500 g for 20 seconds to remove the supernatant. The pellet was then suspended in NMG+/K+ buffer (5 mM HEPES, 10 mM HCl, 1 mM KCl, 200 μM K+ BAPTA, adjusted with N-methyl-D-glutamine (NMG) to pH 5.6 containing 1 mM sulfinpyrazone) containing 1 mM sulfinpyrazone (Sigma-Aldrich, USA) and 3 μM Fura-2 dissolved in DMSO (Sigma Aldrich, USA). Following incubation for 2 h at 36 °C, the suspended sample was washed three times using TAP media. The dye-free NMG+/K+ buffer (pH 6.8) was then added and incubated for 10 minutes at 4 °C in the dark. Finally, Fura-2-Ca^2+^ in the compressed cell image was identified using a confocal laser microscope (LSM 510 META, Carl Zeiss, Germany).

### Sulpho-phospho-vanillin method (colorimetric method)

Samples of *C. reinhardtii* from the reactor were centrifuged at 13,500 g for 20 seconds to remove the supernatant. The pellet was then suspended in 1 ml of 95% ethanol (Samshun Pure Chemical, Republic of Korea) overnight, after which it was centrifuged at 13,500 g for 10 minutes. Next, 100 μl of supernatant were transferred to a 96-well plate and evaporated at 90 °C. This was followed by the addition of 100 μl sulfuric acid into each well and incubation at 90 °C for 20 minutes. Subsequently, 50 μl vanillin-phosphate (0.25 mg/ml vanillin in 17% phosphoric acid) (Sigma Aldrich, USA) was added and incubated at room temperature for 10 minutes. Finally, the optical density was measured at 540 nm using a spectrophotometer (Sunrise^TM^, Tecan, Switzerland).

### GC-MS analysis

*C. reinhardtii* (1 × 10^5^ cell/ml) samples from the reactor were centrifuged at 13,500 g for 20 seconds to remove the supernatant. The pellet was then re-suspended in 1.0 ml of toluene (Junsei Chemical, Japan) and 10% (v/v) acetyl chloride in methanol (Sigma Aldrich, USA) was added. The sample was subsequently incubated for 120 minutes at 70 °C. After cooling the sample, 0.2 ml of distilled water was added to terminate the reaction. Lipids were extracted by adding 1.25 ml of hexane-methyl tert-butyl ether (1:1) (Sigma Aldrich, USA), tumbling gently for 10 minutes, centrifuging the tube at 2500 × g for 6 minutes and then transferring the organic (upper) phase to a fresh tube. Samples were then cleaned by adding 3 ml of cleaning reagent (10.8 g NaOH in 900 ml dH_2_O) and tumbling for 5 minutes. The organic (upper) phase was subsequently transferred to a fresh tube again and evaporated at 40 °C. The FAMEs were then re-suspended in hexane and injected into an Agilent 6890N gas chromatograph equipped with a J&W GC column DB-5ms (30 m × 0.25 mm × 0.25 μm) and a LECO Pegasus III Time-of-Flight Mass Spectrometer (TOMS) as a detector (Agilent Tech, USA). The injection volume was 1 μL with a 10:1 split at an inlet temperature of 280 °C and the carrier gas was helium applied with a fixed flow of 1 mL/min throughout the temperature program, which was as follows: 30 °C for 10 minutes, followed by an increase at 10 °C/min to 300 °C, where they were held for 10 minutes. GC analysis of FAMEs was conducted by the Korea Institute of Science and Technology (KIST) Advanced Analysis Center (ACC).

### Qualitative analysis of lipids in *C. reinhardtii* by Nile red staining

Nile Red (Sigma-Aldrich, USA) staining was performed to observe the lipid accumulation in response to mechanical stress. Samples extracted from the reactor were fixed with 2.5% glutaraldehyde overnight, after which the sample was mixed with 2 μg/ml Nile red dye at a 3:1 ratio and incubated for 10 minutes at 40 °C in the dark. Images of lipid bodies in the compressed cells were obtained by confocal microscopy (LSM 510 META, Carl Zeiss, Germany) at an excitation wavelength of 543 nm and an emission wavelength of 630 nm.

### Quantitative real-time PCR (qRT-PCR)

Real time q-PCR was conducted to measure the expression of mRNA for quantitative analysis of lipid accumulation. *C. reinhardtii* samples from the reactor were centrifuged at 13,500 g for 3 minutes to remove the supernatant, after which cells were counted using a hemocytometer (Mariendeld-Superior, Germany). Each sample was diluted to 5 − 6 × 10^6^ cells/ml based on the results of cell counting. After washing with PBS, samples were centrifuged at 10,000 rpm for 15 seconds. Next, supernatant was removed from the sample, RNA was extracted by RNAiso plus (Takara, Japan) and gDNA was eliminated and total RNA collected using an RNeasy Plant Mini Kit (Qiagen, Germany). The cDNA synthesis of the sample was performed using a Reverse Transcription Kit (Qiagen, Germany) to prevent degradation of the RNA. Gene amplification of the sample was then performed using a QuantiFast SYBR Green PCR Kit (Qiagen, Germany) and Rotor-Gene Q (Qiagen, Germany).

### Statistical analysis

All experiments were statistically carried out in triplicate and significant differences between groups were identified by t-tests. P values (*P < 5.0 × 10^−2^, **P < 1.0 × 10^−2^, ***P < 1.0 × 10^−3^) were considered to indicate significance.

## Additional Information

**How to cite this article**: Cho, M. K. and Shin, H. S. Mechanotransduction-Induced Lipid Production System with High Robustness and Controllability for Microalgae. *Sci. Rep.* **6**, 32860; doi: 10.1038/srep32860 (2016).

## Supplementary Material

Supplementary Information

## Figures and Tables

**Table 1:** GC analysis of lipid contents in *C. reinhardtii* under microbead compression.

	Control	4h-0ml	4h-4ml	8h-0ml	8h-4ml	12h-0ml	12h-4ml
C14:0	5.78	5.46	8.26	4.77	4.41	5.30	4.49
C16:0	27.38	26.6	22.3	23.28	25.26	24.59	32.68
C18:0	23.73	17.5	21.46	17.42	16.57	20.07	19.54
C18:2	0.00	1.37	1.22	0.83	0.91	1.27	1.09
C18:3	0.00	1.98	1.43	1.11	1.12	1.42	1.64
C20:0	10.94	15.52	9.77	12.44	14.74	9.61	8.04
ΣPUFAs	0.00	3.35	2.64	1.94	2.02	2.69	2.73
ΣOthers	32.17	31.58	35.57	40.14	37.0	37.74	32.53
