# Supplementary material for: Mechanotransduction-Induced Lipid Production System with High Robustness and Controllability for Microalgae
Source: Sci Rep. 2016 Sep 9;6:32860. doi: 10.1038/srep32860 (PMC5016897; doi:10.1038/srep32860)
Supplement: Supplementary Information [file srep32860-s1.pdf]

Mechanotransduction-Induced Lipid Production System with High Robustness and Controllability for Microalgae

Supplementary Information

Myung Kwon Cho, Hwa Sung Shin*

Department of Biological Engineering, Inha University, Incheon, 402-751, Korea

* Corresponding author.

Hwa Sung Shin, hsshin@inha.ac.kr, Tel: 82-32-860-9221, Fax: 82-32-872-4046

This file includes:

6 figures and 2 tables which are supporting our research.

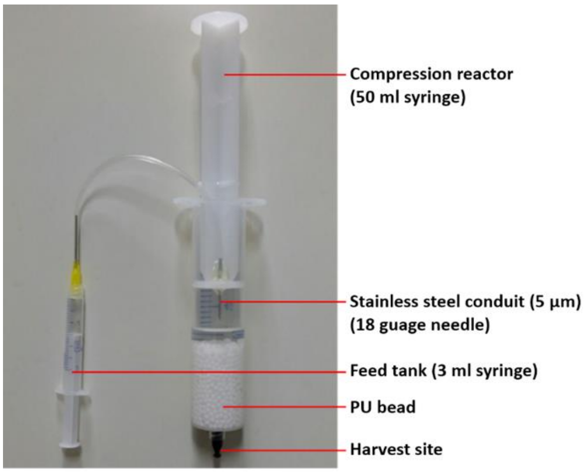

Supplementary Figure S1 Prototype MDLP system using microbeads and syringes.

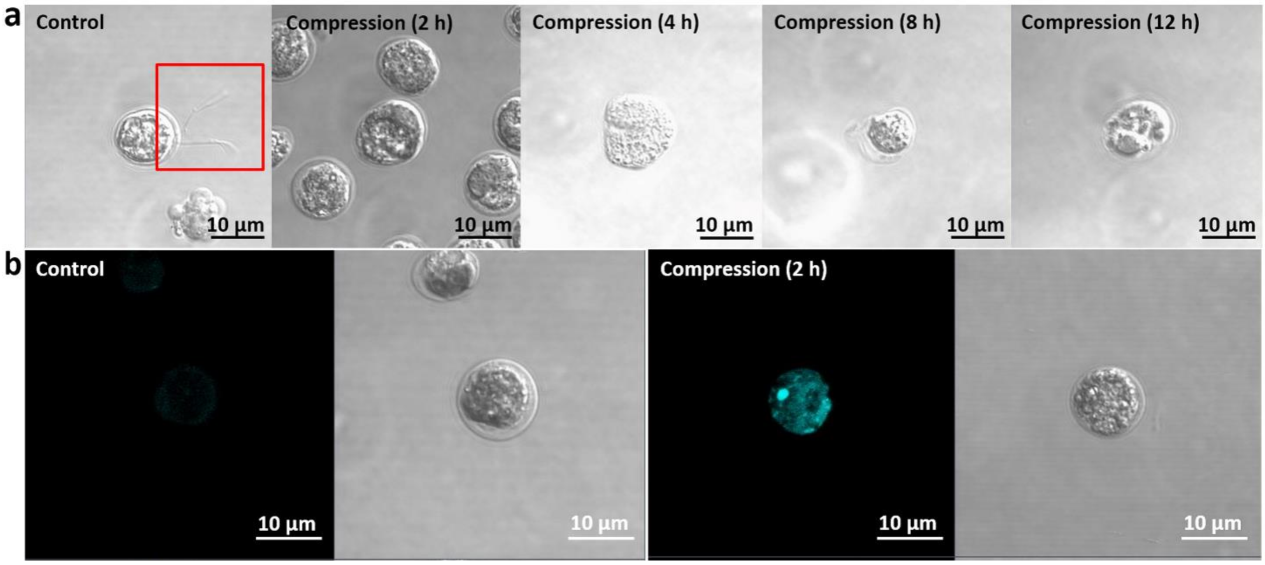

Supplementary Figure S2 Effects of microbeads mechanotransduction on deflagellation and calcium influx of microalgae. (a) Deflagellation of microalgae with respect to compression times. Deflagellation of microalgae was observed in other samples (4, 8, 12 h compression time samples). (b) Calcium influx of *C. reinhardtii* under microbead compression including differential interference contrast (DIC) images of confocal laser microscopy.

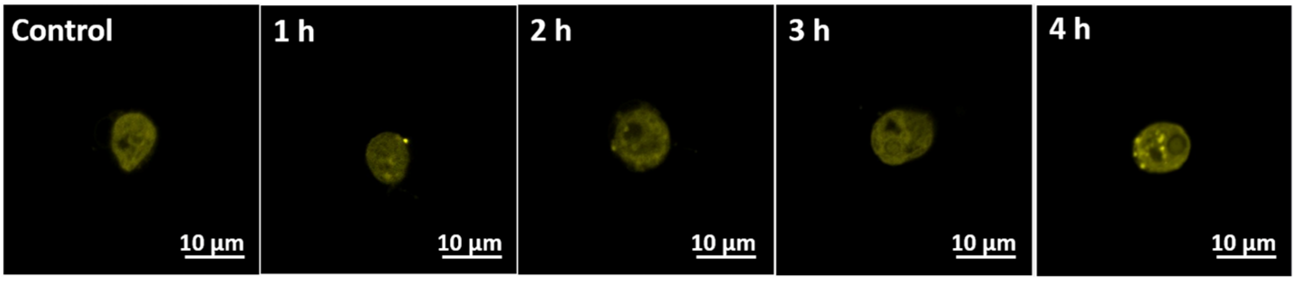

Supplementary Figure S3 Minimum compression time for inducing lipid accumulation in *C. reinhardtii* under microbead compression.

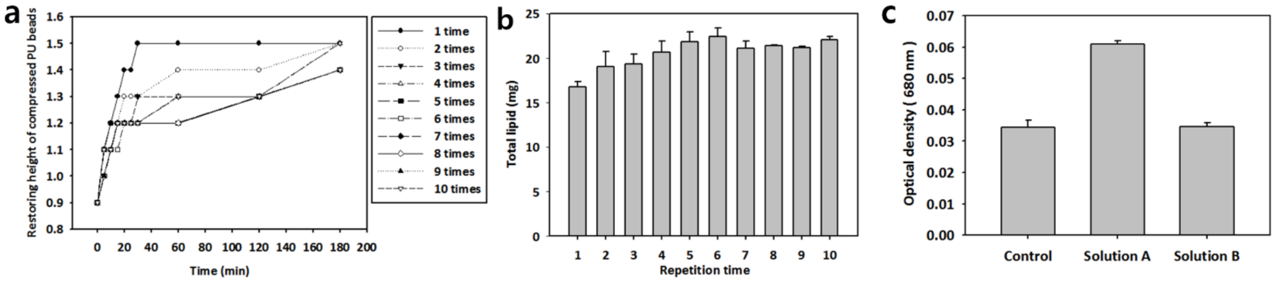

Supplementary Figure S4 Robustness of PU bead under the repeated compression process. (a) The elasticity of bead under the repeated compression process. Same amount of PU beads were compressed at 0.9 cm in 3 ml syringe and the restoring height of compressed PU beads was measured after supplying sufficient TAP media to the syringe. (b) The lipid productivity of MDLP system under the repeated compression process. The repeated compression condition was 8 h compression time and 4 ml de-compression degree. (c) Cytopermeability test of PU bead under bead compression condition. The control solution was TAP media, the solution A was washing liquid that was from the surface of PU bead and the solution B was extracted from inside the PU bead.

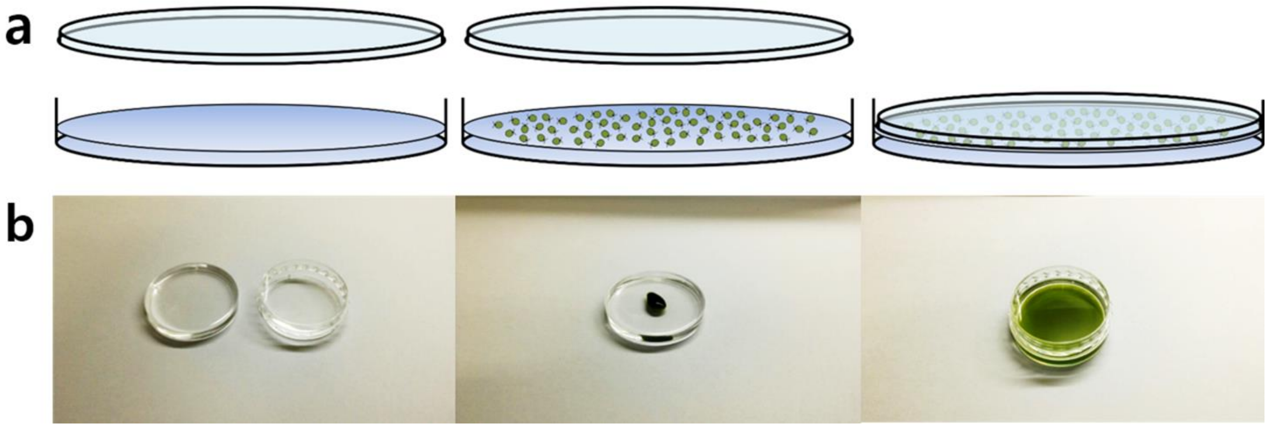

Supplementary Figure S5 Direct compression of *C. reinhardtii*. (a) Scheme of direct compression process. (b) *C. reinhardtii* was compressed by using a 35Ø petri dish. A lid of petri dish was filled with PDMS.

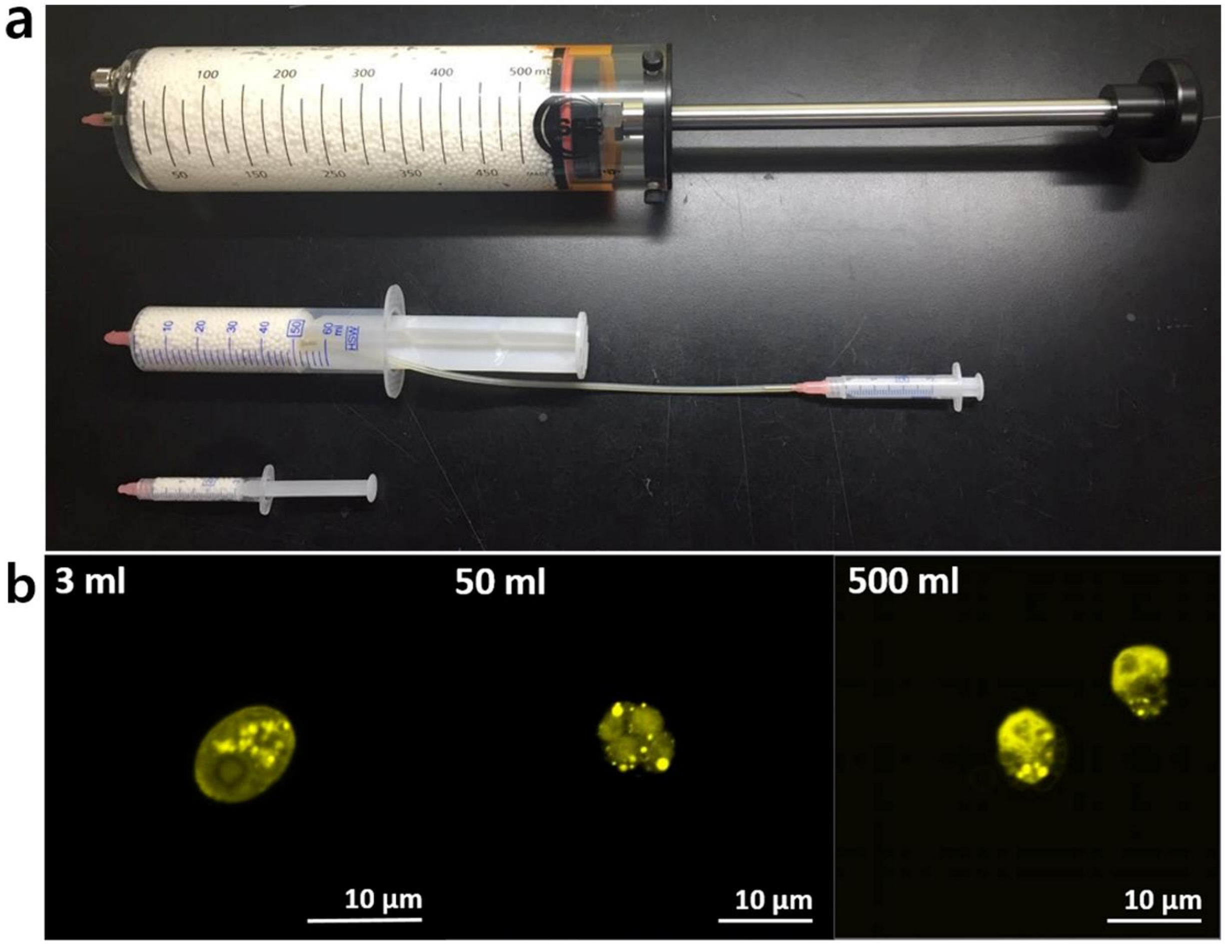

Supplementary Figure S6 Scale-up of MDLP system. (a) Prototype of MDLP system using 3, 50 and 500 ml of syringe. (b) Nile red staining of lipid droplets in microalgae with respect to reactor size.

C. reinhardtii strain	Stress type	Analysis Method	Increase rate (fold)	Process time (h)	Productivity	Reference
CC-621	Bead compression	Sulfo-phospho-vanillin assay	1.79	8	22.375	-
CC-124	Nitrogen deprivation	GC-FID	3.8	96	3.958	[1]
CC-124	Nitrogen deprivation	Extraction by Bligh and Dyer	2.2	96	2.292	[2]
CC-125	Nitrogen deprivation	Extraction by Bligh and Dyer	3.1	96	3.229	[2]
CC-125	Nitrogen deprivation	HPTLC densitometry	2.5	48	5.208	[3]
CC-124	Nitrogen deprivation	HPTLC densitometry	2.1	48	4.375	[3]

[1] Victoria, H. W., et al. Increased Lipid Accumulation in the Chlamydomonas reinhardtii sta7-10 Starchless Isoamylase Mutant and Increased Carbohydrate Synthesis in Complemented Strains. *Eukaryotic cell* **9**, 1251-1261 (2010)

[2] Cakmak, T., Anqun, P., Demireay, YE., Ozkan, AD., Elibol, Z. & Tekinay, T., Differential effects of nitrogen and sulfur deprivation on growth and biodiesel feedstock production of Chlamydomonas reinhardtii. *Biotechnol Bioenq*. **109**, 1947-1857 (2012)

[3] Magali, S., et al. Oil accumulation in the model green alga Chlamydomonas reinhardtii: characterization, variability between common laboratory strains and relationship with starch reserves. *BMC Biotechnology* **11**, http://www.biomedcentral.com/1472-6770/11/7

Supplementary Table S1 Comparison of lipid productivity in accordance with different stress types of systems. The productivity was calculated by lipid increase rate (fold) per process time (h).

Gene	Forward primer sequence (5'-3')	Reverse primer sequence (5'-3')
**CBLP**	CTACGTCAACACCGTGACCG	CATGGCAATGCCGTCCTT
**Mat3**	CGCGAGATATCCGGTCAT	CCAAGTGCAGAGCAAATCATC
**E2F1**	GCAGCCACACAGGAGGTT	CGGCTTGGTTGAGGTCAAGG
**ACCase**	CACAGACGGGTAGTTGTGGA	GAGCTACCTAAGCGCTCCTG
**DGAT**	CCCGGAGGGCACGTGTAT	CCGCGACAGCTAGGTTCCA
**LPAAT**	CGTGATGATAAACCGTGTGG	TCACCCTCAGGGAAGAACAG

Supplementary Table S2 Six pairs of mRNA primer sequence related with cell cycle, apoptosis and lipid synthesis in *C. reinhardtii*
